# Prenatal Diagnosis of Fetus With Transaldolase Deficiency Identifies Compound Heterozygous Variants: A Case Report

**DOI:** 10.3389/fgene.2021.752272

**Published:** 2022-02-04

**Authors:** Jiaxin Xue, Jin Han, Xiaopeng Zhao, Li Zhen, Shanshan Mei, Zhiyang Hu, Xiuzhen Li

**Affiliations:** ^1^ Prenatal Diagnosis Center, Guangzhou Women and Children’s Medical Center, Guangzhou, China; ^2^ Department of Obstetrics and Gynecology, Guangzhou Medical University, Guangzhou, China; ^3^ Division of Neonatology, Guangzhou Women and Children’s Medical Center, Guangzhou, China; ^4^ Division of Obstetrics, Guangzhou Women and Children’s Medical Center, Guangzhou, China; ^5^ Shenzhen People’s Hospital, Shenzhen, China; ^6^ Division of Endocrinology, Guangzhou Women and Children’s Medical Center, Guangzhou, China

**Keywords:** Transaldolase deficiency, pentose phosphate pathway, *TALDO1*, prenatal diagnosis, whole-exome sequencing (WES)

## Abstract

Transaldolase (TALDO) deficiency is a rare autosomal recessive disorder caused by variants in the *TALDO1* gene that commonly results in multisystem dysfunction. Herein, we reported compound heterozygous variants in a Chinese prenatal case with TALDO deficiency using whole-exome sequencing (WES) for trios and Sanger sequencing. The heterozygous variants were located on the *TALDO1* gene: NM_006755.2:c.574C > T(Chr11:g.763456C > T), a missense variant in exon 5 paternally inherited; NM_006755.2:c.462-2A > G(Chr11:g.763342A > G), a splicing aberration in intron 4 maternally inherited. The qualitative analysis of urinary polyols in neonatal urine indicated that xylitol + arabitol and ribitol in the proband’s urine were significantly increased. These findings expand the variation spectrum of the *TALDO1* gene, provide solid evidence for the counseling of the family in regard to future pregnancies, strongly support the application of WES in prenatal diagnosis, and further prove that effective postpartum treatments could improve prognosis.

## Introduction

Transaldolase (TALDO) deficiency (OMIM 606003), a rare metabolic congenital defect of the pentose phosphate pathway (PPP), is caused by homozygous or compound heterozygous variants of the *TALDO1* gene (Wamelink et al., 2008) located on chromosome 11p15. Its main clinical manifestations usually appear in the neonatal period, while they are relatively rare in the antenatal period. The typical symptoms include coagulopathy, thrombocytopenia, liver dysfunction, hepatosplenomegaly, hepatic fibrosis, hemolytic anemia, generalized edema, dysmorphic features, and renal dysfunction that rarely occurs. Prolonged activated partial thromboplastin time (APTT), prothrombin time (PT) and thrombin time (TT), low cholesterol, high alkaline phosphatase (AKP), as well as elevated total bilirubin (TBIL), direct bilirubin (DBIL), total bile acid (TBA), and β2-microglobulin (β2-MG), can indicate liver and renal dysfunction in some reported cases (Valayannopoulos et al., 2006).

The PPP has two main functions: 1) It provides reduced nicotine adenine dinucleotide phosphate (the cofactor of redox reaction for organism biosynthesis), and 2) it offers ribose-5-phosphate to the nucleic acid. The PPP is divided into oxidative (nonreversible) and nonoxidative (reversible) enzymatic reactions/parts. Moreover, TALDO is the second enzyme of the nonoxidative part tightly linking the PPP and glycolysis pathway (Verhoeven et al., 2005).

To date, approximately 39 cases diagnosed with TALDO deficiency have been reported, but the incidence is unclear (Verhoeven et al., 2001; Eyaid et al., 2013; Rodan and Berry, 2017; Halabi et al., 2019; Lee-Barber et al., 2019; Williams et al., 2019; Lafci et al., 2021) (Table 3). Yet, the pathophysiology leading to TALDO deficiency remains unclear due to the low number of reported cases. TALDO deficiency can also have high variability in clinical manifestations and outcomes, even within the same family (Tylki-Szymanska et al., 2009; Leduc et al., 2014). Herein, we reported a novel compound heterozygous variant in a Chinese prenatal case with multiorgan dysfunction confirmed as TALDO deficiency by prenatal molecular diagnosis.

## Materials and Methods

### Ethics Approval

After receiving written informed consent from both of the parents, WES (trio analysis of the proband, mother, and father) was carried out. Our study was approved by the Ethics Committee of Guangzhou Women and Children’s Medical Center and Guangzhou Medical University, and it conformed with the ethical standards of experiments on human subjects.

### Case Presentation

A 33-year-old pregnant woman, gravida 2, para 1, was referred to our hospital at 34 weeks because of ultrasonic abnormalities. Fetal middle cerebral artery peak systolic velocity (MCA-PSV) kept increasing from 24 gestational weeks, reaching 93.97 cm/s [>1.5 MoM (Multiples of the Median)] at 33 gestational weeks. Additional anomalies included a slightly high echo of the right lobe of the liver, cardiomegaly with the cardiothoracic ratio of 0.61, a small amount of pericardial effusion, and placental thickness of 46 mm.

Similar manifestations, including cardiac enlargement, hepatosplenomegaly, placental thickness, elevated MCA-PSV, high umbilical artery resistance, and intrauterine growth restriction (IUGR), were observed during the first pregnancy (II:1). Following fetal distress, at 36 weeks, a baby boy was born by cesarean section weighing 1,860 g (<10th). His Apgar scores were normal (9′-10′-10′), while the neonatal peripheral blood test detected that hemoglobin (HGB) and platelet (PLT) were low. Repeated examinations of coagulation showed extended APTT, PT, and TT. Brain ultrasound suggested a head injury with subependymal hemorrhage. Therefore, II:1 received human immunoglobulin and blood transfusion to prevent infection and improve blood coagulation. The neonate did not recover and consequently died of disseminated intravascular coagulation (DIC), a low-birth-weight, and hypoproteinemia at 18 days. Clinical findings of the two affected fetuses (II:1 and II:2) are summarized in Tables 1, 2.

**TABLE 1:** Ultrasound findings of II:2 at different gestational weeks.

Test time(weeks)	NT/NF^a^ (mm)	MCA-PSV^b^ (cm/s)	Reference interval of MCA-PSV (cm/s)^c^	Cardiothoracic ratio	Pericardial effusion (mm)	Placenta thickening (mm)	Right lobe length of liver (mm)	Reference interval of liver (mm)^d^
12+	1.1	–	–	–	–	16	–	–
17+	4.9	–	–	–	–	22	–	–
22+	–	–	–	<0.50	–	26	–	–
25+	–	44.0	23.6–40.8	<0.50	–	32	–	–
27+	–	56.7	26.8–46.1	<0.50	–	33	–	–
33+	–	93.0	36.0–46.3	0.57	2.8	46	57.7	40.6–52.3
37+	–	93.9	38.9–75.4	0.64	4.0	48	64.0	47.0–58.7

a Note. NT, nuchal translucency (normal <3.0 mm); NF, neck fold (normal <6.0 mm).

b MCA-PSV, middle cerebral artery peak systolic velocity.

c Ebbing et al., 2007.

d Tongprasert et al., 2011.

**TABLE 2:** The laboratory results of II:2 as determined directly in 24+ and 28+ weeks through the umbilical cord blood tests and after birth between II:1 and II:2.

Time test	II:2^a^			II:2	II:1^b^	Reference interval
	24 Gestational weeks	28 Gestational weeks	Reference interval	Newborn	Newborn	
TT^c^(s)	–	–	–	23.6	34.8	14–21
PT(s)	–	–	–	30.7	31.7	11–15
APTT(s)	–	–	–	80.5	124.7	28–45
AKP(U/L)	289	359	15–121	354	-	118–390
TBIL(µmol/L)	23.1	39.3	1.7–20.0	83.3	157.52	2–17
DBIL(µmol/L)	2.76	3.89	0–6	10.0	96.3	0–7
TBA(µmol/L)	–	–	–	18.4	75.41	0.5–10.0
β2-MG (mg/L)	10.85	4.71	0.7–1.8	–	–	–
HGB (g/L)	82	104	110–150	95	104	135–195
PLT (*10^9/L^)	123	137	100–300	79	48	140–440
HbA (%)	4.0	5.3	96.8%–97.8%	–	–	–
LDH (U/L)	263	256	110–240	989	1,679	159–322
AST (U/L)	24	0–45	26	89	63	5–60
ALB (g/L)	–	–	–	23.1	16.5	40–55

a Note. The second offspring.

b The first offspring.^c^TT, thrombin time; PT, prothrombin time; APTT, activated partial thromboplastin time; AKP, alkaline phosphatase; TBIL, total bilirubin; DBIL, direct bilirubin; TBA, total bile acid; β2-MG, β2-microglobulin; HGB, hemoglobin; PLT, platelet; HbA, hemoglobin A; LDH, lactate dehydrogenase; AST, aspartate aminotransferase; ALB, albumin.

**FIGURE 1:**
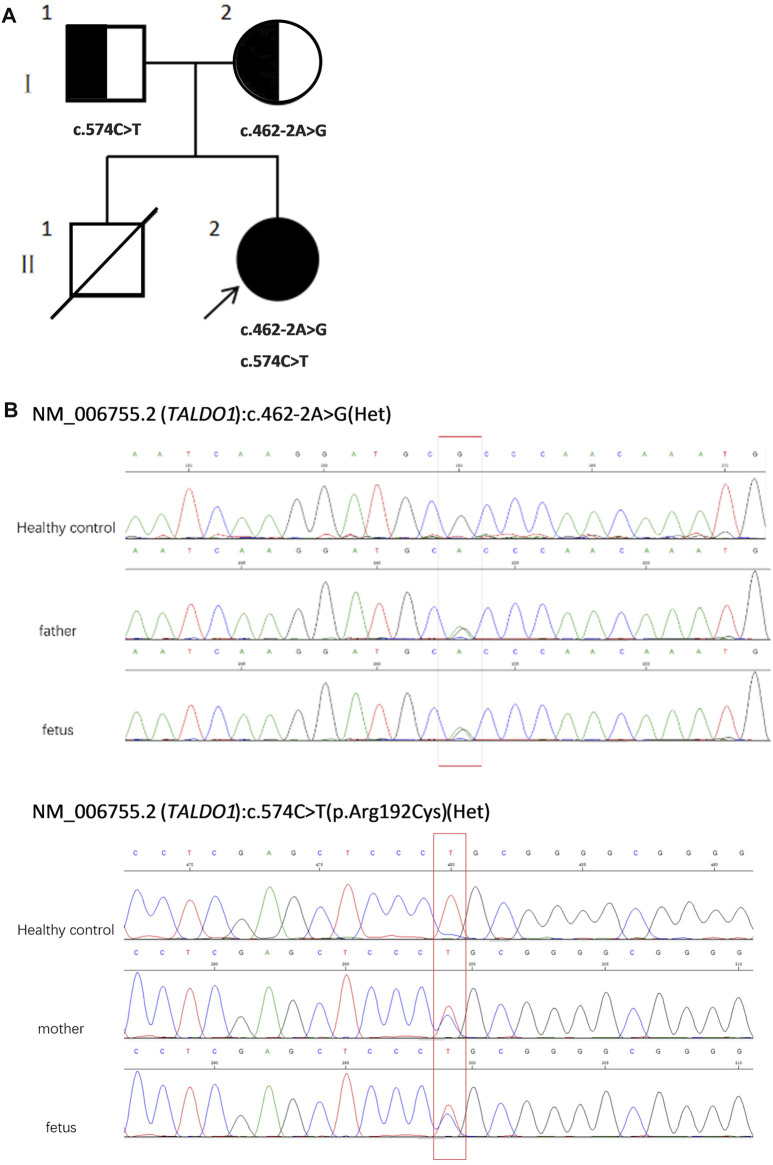
**(A)** Pedigree of the family. **(B)** Sequencing of *TALDO1* gene (reference cDNA sequence, NM_006755.2) revealed two heterozygous variations, resulting in A to G splicing at nucleotide position 462–2 (c.462-2A > G) and C to T substitution at nucleotide position 574 [c.574C > T(p.Arg192Cyrs)]. Het, heterozygous.

To assess the risk of recurrence, cordocentesis was performed for genetic diagnosis, including karyotype analysis and chromosomal microarray analysis (CMA), to clarify the potential cause of the disease two times in another hospital, but the results were negative. Consequently, WES was performed on the proband and his healthy parents (Figures 1A,B) to search for potential variants. The detailed examinations during pregnancy are listed in Table 1.

### Metabolite Analyses

Urine xylitol + arabitol and ribitol were measured using gas chromatography-mass spectrometry (GC-MS). Urine sample preparation was based on urease pretreatment methods. Samples were standardized to 0.25 mg creatinine. Derivatization was performed with 100 μl bis-(trimethylsilyl) trifluoracetamide + 1% trimethylchlorosilane and was allowed to react at 60°C for 10 min. The metabolites were chromatographically analyzed as trimethylsilyl compounds.

### Whole-Exome Sequencing

Genomic DNA was randomly fragmented and purified using the magnetic particle method. WES was performed on an IIIumina HiSeq 2,500 sequencer (Illumina, San Diego, CA, United States) for a minimal of 10.14 Gb read-depth per case. Sequencing reads after quality control were aligned to the human reference genome by BWA (hg19). Nucleotide changes of aligned reads were reviewed using NextGENe software (Version 2.4.1.2) (SofGenetics, State College, PA, United States). Novel variants were filtered against the 1,000 Genomes database (http://www.1000genomes.org/), dbSNP database (http://www.ncbi.nlm.nih.gov/projects/SNP/snp_summary.cgi), and the Genome Aggregation database (gnomad.broadinstitute.org). Databases, including ClinVar (version: #372716), OMIM (version: #602063.0005), ClinGen (version: #CA5788214), and Human Gene Mutation database, were used. In addition, software (SIFT, Polyphen, MutationTaster, PROVEAN and REVEL) was used to predict the impact of missense variants. For the splicing variant, the *in silico* prediction tools were dbscSNV and MaxEntScan. Common variants (with high minor allele frequency in normal population; gnomAD) were eliminated. Finally, polymerase chain reaction (PCR) was performed to amplify the affected fragment of *TALDO1* gene using specific primers, and the purified PCR products were applied to Sanger sequencing to affirm the variant(s).

## Results

**FIGURE 2:**
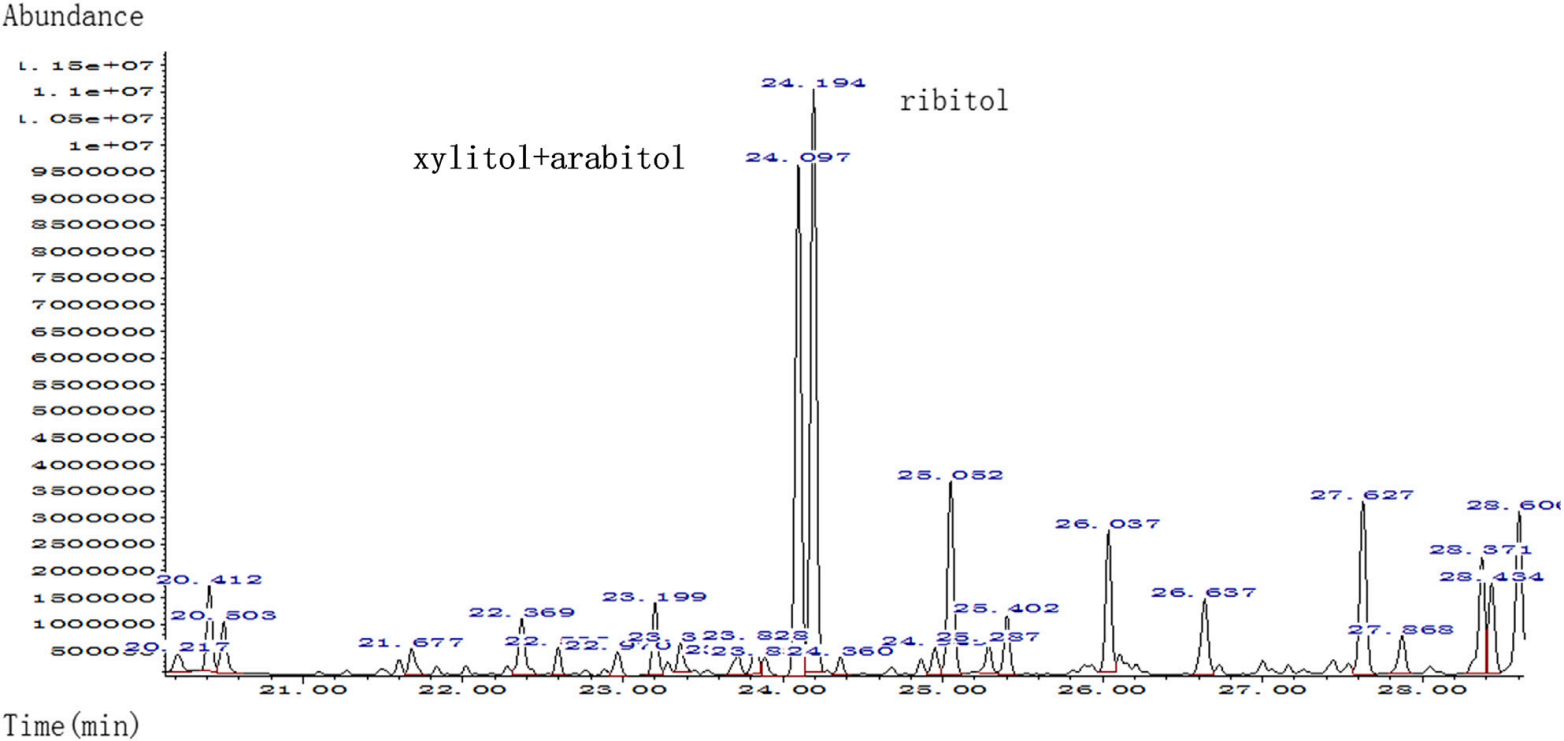
Quality analysis of polyols in urine sample of patient by urine gas chromatography-mass spectrometry (GC-MS); markedly elevated xylitol + arabitol and ribitol were detected.

The umbilical cord blood samples of the fetus (II:2) in 24 and 28 gestational weeks in another hospital showed fetal anemia, thrombocytopenia, coagulation dysfunction, and elevated liver enzymes [lactate dehydrogenase (LDH) and β2-MG] **(** Table 2 **)**. Urine test for metabolic compounds using GC-MS showed an elevation of xylitol + arabitol at 170,388 mmol/mol creatinine (normal 0–1,151 mmol/mol creatinine) and ribitol at 193,301 mmol/mol creatinine (normal 0–886 mmol/mol creatinine) (Figure 2).

WES revealed compound heterozygosity of variants in the *TALDO1* gene in the proband: maternally inherited likely splicing aberration NM_006755.2(*TALDO1*):c.462-2A > G(Chr11:g.763342A > G), and paternally inherited missense variant NM_006755.2(*TALDO1*):c.574C > T(Chr11:g.763456C > T). Both parents were heterozygous carriers and phenotypically normal. Sanger sequencing of the patient and his family members further validated these results (Figures 1A,B). According to the ACMG standards, both variants were defined as likely pathogenic (c.462-2A > G: PVS1 + PM2, c.574C > T: PM2 + PM3 + PM5 + PP3). The two variants had a very low carrying rate in some databases: c.462-2A > G is not recorded in gnomAD, dbSNP, or 1,000 Genomes databases; the minor allele frequency of c.574C > T is .00001591 (4 heterozygotes) in gnomAD, and 0.000008243 (1 heterozygote) in ExAC. c.574C > T is a missense variant located in exon 5, which is also described on dbSNP (rs751425603) and reported as pathogenicity in ClinVar (variation ID: 381,759). c.462-2A > G is a splicing variant located in intron 4 and may lead to abnormal mRNA splicing that affects protein expression. The splicing variant c.462-2A > G in MaxEntScan score was from 10.76 in wild type to 2.824 in mutant type. This variant is predicted to cause a loss of function of the protein.

## Outcome

**FIGURE 3:**
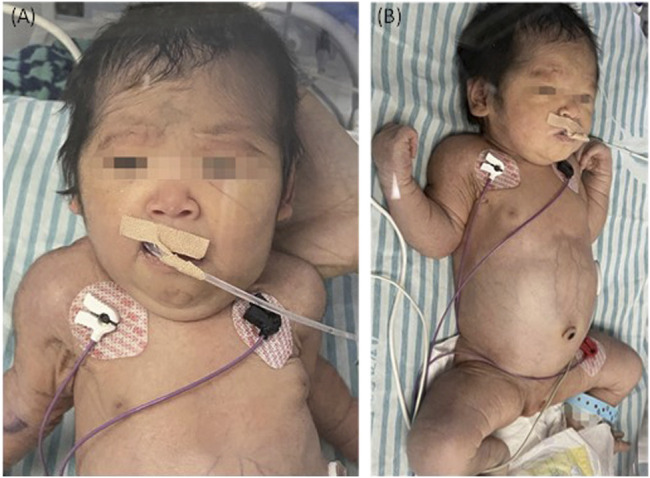
The TALDO-deficient dysmorphic feature-hirsutism (forehead), low hair implantation, mild pallor, and cuties laxa with visible vascular network of the patient. The abdomen was also grossly distended with dilated visible veins [**(A)** frontal view, **(B)** side view].

The couple decided to continue the pregnancy after genetic counseling. A baby girl was born at 38 weeks, with a weight of 2,760 g. Apgar scores were normal (8′-8′-8′) after delivery. At birth, the baby had dysmorphic features (hirsutism, low hair implantation), mild pallor, and cutis laxa. She also presented low skin temperature, quick breath with groaning, thick breath sounds in both lungs with moist rales, and abdominal distention with the visible vascular network. The baby was hospitalized at the Neonatal Department in our hospital for 9 days (Figures 3A,B). She had hepatosplenomegaly and developed jaundice. A peripheral blood test showed HGB of 95 g/L (normal 110–150 g/L) with fragmented red cells on film, thrombocytopenia, and mild neutropenia, consistent with those *in utero*. Serum TBIL was 83.3 µmol/L (normal 2–17 µmol/L) with DBIL of 10.0 µmol/L (normal 0–7 µmol/L). LDH was also increased to 989 U/L (normal 159–322 U/L) together with marginally elevated transaminases, bile acids and alkaline phosphatase (ALP). Albumin was 23.1 g/L (normal 40–55 g/L) and PT was 31.7 s (normal 11–15 s). The infant received continuous ventilation for 9 days. Fresh frozen plasma and fibrinogen infusion were given to improve thrombocytopenia and coagulation. Blood glucose level was stable and was closely monitored. GC-MS indicated elevated urinary xylitol + arabitol and ribitol levels.

After her condition gradually improved, the patient was discharged from the hospital and was regularly followed up. At the age of 9 months, HGB was still slightly decreased (100 g/L), while red blood cell and PLT were both increased to 4.7 × 10^12/L^ (normal 3.5–5.0 × 10^12/L^) and 517 × 10^9/L^ (normal 100–300 × 10^9/L^), respectively. DBIL, TBIL, TBA, LDH, and AKP levels were normal, whereas aspartate aminotransferase (AST) mildly elevated to 84 U/L. The dysmorphic features and cutis laxa were not observed. Thus far, the child has shown normal physical and cognitive development.

## Discussion

TALDO deficiency is a rare autosomal recessive error of the PPP caused by a variant in the *TALDO1* gene (Williams et al., 2019). TALDO1 gene encodes TALDO implicated as a major modulator between the PPP and glycolysis in a reversible reaction. TALDO catalyzes the conversion of glyceraldehyde-3-phosphate and sedoheptulose-7-phosphate into fructose-6-phosphate and erythrose-4-phosphate, which are also considered targets for the treatments of this condition. In addition, its absence can result in the accumulation of intermediate products (e.g., sedoheptulose, erythritol, and ribitol) and eventually cause lesions of the patent. TALDO deficiency has been associated with a range of phenotypes, including intrauterine lethality together with fetal multimalformation syndrome and hydrops fetalis. The most common clinical manifestations in neonates are cirrhosis, liver failure, hepatosplenomegaly, anemia, thrombocytopenia, dysmorphia, congenital heart defects, and tubulopathy (Verhoeven et al., 2001; Verhoeven et al.,2005; Valayannopoulos et al., 2006; Wamelink et al., 2008; Tylki-Szymanska et al., 2009; Balasubramaniam et al., 2011; Eyaid et al., 2013). Yet, prenatal diagnosis is very challenging. Abnormal findings in the fetus are rare. Some of the common manifestations in the antenatal period are IUGR (Verhoeven et al., 2001; Valayannopoulos et al., 2006; Wamelink et al., 2008), oligohydramnios, fetal splenomegaly, fetal distress (Wamelink et al., 2008), and hyperechogenic bowel (Banne et al., 2015). Also, TALDO deficiency can be easily misdiagnosed with gestational alloimmune liver disease (GALD). GALD is the result of maternal alloimmune injury, which includes neonatal liver failure (coagulation disorders, ascites, and hypoalbuminemia), intrahepatic, and extrahepatic iron accumulation (hemosiderosis).

In this study, a family that had experienced neonatal death following IUGR, hepatosplenomegaly, anemia with thrombopenia, and abnormal coagulation tests in a previous pregnancy (II:1) and recurrent fetal anemia, hepatosplenomegaly in the second pregnancy (II:2), was recruited for WES. A prenatal diagnosis of the fetus confirmed heterozygous variants in the *TALDO1* gene in II:2. Yet, prenatal findings were different between II:1 and II:2. Fetal MCA-PSV increased from 24 gestational weeks, which reflected fetal anemia *in utero*. Additional ultrasound anomalies identified at 33 gestational weeks included slightly high echo of the right lobe of the liver, cardiomegaly with increased cardiothoracic ratio, a small amount of pericardial effusion and placental thickness, all of which suggested a progressive development of fetal anemia. After birth, the postpartum symptoms were clearer and more obvious, including dysmorphic features, liver dysfunction and hemolytic anemia. This case is consistent with the range of phenotypes most commonly observed; however, fetal anemia, liver dysfunction, and coagulopathy are the main manifestations.

The accumulation of sugars and polyols [e.g., sedoheptulose-7-phosphate, ribose-5-phosphate, ribulose-5-phosphate, xylulose-5-phosphate, and C5-polyols (i.e., D-ribitol and D-arabitol)] are believed to be the cause of liver involvement in TALDO deficiency. Higher concentrations of the polyols xylitol + arabitol and ribitol in the urine of the proband could be relevant of the phenotypes in II.2, but could also be related to the younger age, since the polyol concentrations were higher in the neonatal period in other patients and accumulated less when they were older (Wamelink et al., 2008). Although from the same family, patient II:1 had IUGR, anemia, hepatosplenomegaly, DIC, a low-birth-weight, and secondary hemorrhage (subependymal hemorrhage), yet, even considering that molecular analysis was not performed for patient II:1, it was likely that these phenotypes were associated with TALDO deficiency.

To the best of our knowledge, this case is the first prenatal diagnosis of TALDO deficiency in a Chinese population (Verhoeven et al., 2001; Eyaid et al., 2013; Rodan and Berry, 2017; Lee-Barber et al., 2019; Williams et al., 2019; Halabi et al., 2019; Lafci et al., 2021). Both variants of this case were defined as likely pathogenic. One of the variants [c.574C > T p.(Arg192Cys)], reported as pathogenicity in ClinVar (Variation ID: 381,759), was previously reported in an Arab patient, suggesting a founder effect in Arab populations (Wamelink et al., 2008). The other is a novel splicing variant (c.462-2A > G), which is predicted to affect splicing while not exon skipping. The in-silico tools are dbscSNV and MaxEntScan. They all predict altering *TALDO1* exon splicing. To date, there have been 13 variants reported to cause this condition worldwide (Table 3). Individuals with the same variant show different clinical manifestations.

**TABLE 3:** Summary of clinical manifestations in the current patients with TALDO deficiency.

Case	Variant	Gender	Ethnicity	Consanguinity	Pregnancy	Dysmorphism	Liver dysfunction	Hepatosplenomegaly	Anemia	Thrombocytopenia	Impaired coagulation	Cardiac abnormalities	Neonatal edema	Renal	Respiratory	Developmental delay	Abnormal genitalia	Clinical course
**1** ^**a**^	**NM_006755.2:c.512_514del**	F	Turkey	**+**	IUGR	**-**	**+**	**+**	**+**	**+**	**+**	Aortic	**-**	**-**	**-**	**+**	**+**	Hepatosplenomegaly, telangiectasias of her skin, enlarged clitoris
												coarctation						
**2** ^**b**^ *	**NM_006755.2:c.575G > A**	F	Turkey	**+**	HELPP syndrome	**+**	**+**	**+**	**+**	**+**	**+**	Cardiomyopathy	**+**	Glomerular	**+**	**-**	**-**	*
												large venous		proteinuria				
												duct						
**3** ^**c**^ *	**NM_006755.2:c.512_514del**	F	Turkey	**+**	***n***	**+**	**+**	**+**	**+**	**+**	**+**	ASD, MVP	**-**	Nephrocalcinosis	**-**	**-**	**-**	*
**4** ^**c**^ * (fetus)	**NM_006755.2:c.512_514del**	M	Turkey	**+**	***n***	**+**	**+**	- (Splenic fibrosis)	**+**	**-**	**-**	Cardiomegaly	**+**	-	**-**	**-**	**-**	*
**5** ^**c**^	**NM_006755.2:c.512_514del**	M	Turkey	**+**	***n***	**+→*n***	**+**	**+**	**+**	**+**	**+**	PFO	**-**	Chronic renal	**-**	**-**	**-**	**n/-**
														failure				
														hypoplastic				
														kidney				
**6** ^**c**^	**NM_006755.2:c.512_514del**	M	Turkey	**+**	Oligohydramnion	**+→n**	**+**	**+**	**+**	**+**	**+**	Cardiomegaly	**-**	Transient renal	**-**	**-**	**-**	**n**
					splenomegaly							PFO		failure				
					fetal distress													
**7** ^**d**^	**NM_006755.2:c.574C > T**	M	Arab	**+**	IUGR	**+**	**+**	**+**	**+**	**+**	**+**	Small patent	**-**	Tubulopathy	**-**	Mild delay	**-**	Speech delay (deaf)
												ductus						
**8** ^**e**^	**NM_006755.2:c.575G > A**	M	Pakistani	**+**	***n***	**+→n**	**+**	**+**	**-**	**+**	**+**	**-**	**-**	**-**	**-**	Mild delay	**-**	Speech delay
**9** ^**f**^	**NM_006755.2:c.575G > A**	M	Poland	**+**	***n***	**-**	**+**	**+**	**+**	**+**	**+**	**-**	**-**	**-**	**-**	**-**	**-**	Hepatosplenomegaly
**10** ^**f**^	**NM_006755.2:c.575G > A**	M	Poland	**+**	***n***	**-**	**+**	**+**	**+**	**+**	**+**	**-**	**-**	**-**	**-**	**-**	**-**	Unilateral cryptorchidism, hepatosplenomegaly
**11** ^g*^	**NM_006755.1:c.895_897del; NM_006755.1:c.931G > A**	M	China	**-**	***n***	**+**	**-**	**+**	**+**	**+**	**+**	**+**	**+**	**-**	**+**	**-**	**-**	***n***
**12** ^**h−1**^	**NM_006755.2:c.793del**	F	Saudi Arabia	**+**	A dilated left ventricle	**+**	**+**	**+**	**+**	**+**	**-**	PFO,PDA	**-**	**-**	**-**	**+**	**-**	***n***
**13** ^h−1*^	**NM_006755.2:c.793del**	M	Saudi Arabia	**+**	Polyhydramnios	**+**	**+**	**+**	**+**	**+**	**-**	PFO, ASD	**-**	**-**	**-**	**-**	**-**	*
**14** ^**h−1**^	**NM_006755.2:c.793del**	M	Saudi Arabia	**+**	**n**	**+**	**+**	**+**	**+**	**+**	**-**	+	**-**	**-**	**-**	**+**	**-**	***n***
**15** ^**h−2**^	**NM_006755.2:c.793del**	M	Saudi Arabia	**+**	**—**	**+**	**+**	**+**	**+**	**+**	**-**	PFO, ASD	**-**	**-**	**+**	**-**	**-**	***n***
**16** ^**h−2**^	**NM_006755.2:c.793del**	F	Saudi Arabia	**+**	**—**	**+**	**+**	**+**	**+**	**+**	**-**	PFO	**-**	**-**	**-**	**-**	**-**	***n***
**17** ^**h−2**^	**NM_006755.2:c.793del**	M	Saudi Arabia	**+**	Mild pericardial	**+**	**+**	**+**	**+**	**+**	**-**	PDA, VSDs, and ASD	**-**	**-**	**-**	**-**	**-**	***n***
					effusion, cardiomegaly, and echogenic bowel													
**18** ^**h−3**^	**NM_006755.2:c.793del**	M	Saudi Arabia	**+**	***n***	**+**	**+**	**+**	**+**	**+**	**-**	ASD	**-**	**-**	**+**	**-**	**-**	***n***
**19** ^**h−3**^	**NM_006755.2:c.793del**		Saudi Arabia	**+**	IUGR, oligohydramnios, situs inversus totalis, thick nuchal skin, slightly enlarged right heart, and hepatosplenomegaly	**+**	**+**	**+**	**+**	**+**	**-**	PDA	**+**	**-**	**-**	**-**	**-**	***n***
**20** ^**h−3**^	**NM_006755.2:c.793del**	M	Saudi Arabia	**+**	***n***	**+**	**+**	**+**	**+**	**+**	**-**	ASD	**-**	**-**	**-**	**-**	**-**	***n***
**21** ^**h−4**^	**NM_006755.2:c.793del**	F	Saudi Arabia	**+**	IUGR	**+**	**+**	**+**	**+**	**+**	**-**	**+**	**-**	**-**	**-**	**-**	**+**	***n***
**22** ^**h−5**^	**NM_006755.2:c.793del**	F	Saudi Arabia	**+**	***n***	**-**	**+**	**+**	**+**	**+**	**+**	**+**	**-**	**+**	**-**	**-**	**-**	***n***
**23** ^**h−6**^	**NM_006755.2:c.793del**	F	Saudi Arabia	**+**	***n***	**+**	**-**	**+**	**+**	**+**	**-**	**+**	**-**	**-**	**-**	**-**	**-**	***n***
**24** ^**i**^	—	M	Saudi Arabia	**+**	***n***	**+**	**+**	**+**	**+**	**+**	**-**	PDA, VSDs, and ASD	**-**	**-**	**+**	**-**	**-**	***n***
**25** ^**j**^	**c.462-174_981 + 53del**	M	Poland	**-**	IUGR, ascites, and oligoamnios	**+**	**+**	**+**	**+**	**+**	**-**	**-**	**-**	**+**	**-**	**-**	**-**	***n***
**26** ^**j**^	**NM_006755.2:c.575G > A; c.462-174_981 + 53del**	M	Poland	**–**	IUGR, ascites, and oligoamnios	**+**	**+**	**+**	**+**	**+**	**-**	**-**	**-**	Renal calculus	**-**	**-**	**+**	***n***
**27** ^**k**^	**NM_006755.2:c.512C > T**	M	Gambia	—	***n***	**-**	**-**	**+**	**-**	**-**	**-**	**-**	**-**	**+**	**-**	**-**	**-**	***n***
**28** ^**k**^	**NM_006755.2:c.512C > T**	M	Gambia	—	***n***	**-**	**+**	**+**	**-**	**-**	**-**	**-**	**-**	**-**	**-**	**-**	**-**	***n***
**29** ^**k**^	**NM_006755.2:c.512C > T**	M	Gambia	—	***n***	**-**	**-**	**+**	**-**	**-**	**-**	**-**	**-**	**-**	**-**	**-**	**-**	***n***
**30** ^**l**^	**NM_006755.2:c.574C > T**	M	UAE	**+**	***n***	**-**	**+**	**+**	**+**	**+**	**-**	ASD, PFO	**-**	**-**	**-**	**-**	**-**	***n***
**31** ^**l**^	**NM_006755.2:c.574C > T**	F	UAE	**+**	***n***	**-**	**+**	**+**	**+**	**+**	**-**	**—**	**-**	Proteinuria	**-**	**-**	**-**	***n***
**32** ^**l**^	**NM_006755.2:c.574C > T**	F	UAE	**+**	***n***	**+**	**+**	**+**	**+**	**+**	**-**	LVH, HTN	**-**	Proteinuria	**-**	**+**	**-**	***n***
**33** ^l*^	**NM_006755.2:c.574C > T**	M	UAE	**+**	***n***	**+**	**+**	**+**	**+**	**+**	**+**	RAD, RVH, TR, PDA	**+**	**-**	**-**	**+**	**-**	*
**34** ^m*^ **(fetus)**	**NM_006755.2:c.669C > G**	M	Saudi Arabia	**+**	IUGR, bowel echogenicity	**+**	**+**	+/- (Only splenomegaly)	**-**	**+**	**-**	**+**	**-**	**+**	**-**	**+**	**-**	Acute anisocoria
**35** ^**n**^	**NM_006755.2:c.574C > T**	M	UAE	**-**	***n***	**+**	**+**	**+**	**-**	**+**	**+**	**-**	**-**	**-**	**-**	**-**	**-**	Uncertain
**36** ^**o**^	**NM_006755.2:c.512_514del**	M	United States	—	IUGR	**+**	**+**	**+**	**-**	**+**	**-**	**-**	**-**	**-**	**+**	**-**	**-**	***n***
	**NM_006755.2:c.931G > T**																	
**37** ^**p**^	**NM_006755.2:c.715C > G**	M	Turkey	**+**	—	**+**	**-**	**+**	**+**	**-**	**-**	**-**	**-**	**+**	**-**	**-**	**+**	***n***
**38** ^q*^	**NM_006755.2:c.793del**	F	Saudi Arabia	**+**	***n***	**+**	**+**	+/- (Only splenomegaly)	**+**	**+**	**-**	**-**	**-**	**-**	**+**	**-**	**-**	***n***
**39**	**NM_006755.2:c.574C > T**	F	This case (China)	**-**	Anemia, hepatosplenomegaly, and coagulation dysfunction	**+**	**+**	**+**	**+**	**+**	**+**	**-**	**-**	**+**	**-**	**-**	**-**	***n***
	**NM_006755.2:c.462-2A > G**																	
In addition, there are neonatal hypotonia, intermittent hypoglycemia, characteristic skin vascular changes (hemangioma, spider nevus), and rare diseases such as rickets, sensorineural deafness, and hypothyroidism																		
Total				30/39 (76.9%)	14/39 (35.9%)	30/39 (76.9%)	34/39 (87.2%)>	39/39 (100%)	32/39 (82.0%)	34/39 (87.2%)	14/39 (35.9%)	25/39 (64.1%)	5/39 (12.8%)	14/39 (35.9%)	7/39 (17.9%)	8/39 (20.5%)	4/39 (10.2%)	

Note. +, present; -, not present; n, normal; *, patient died;/not mentioned; →, change to; ASD, atrium septum defect; PFO, patent foramen ovale; MVP, mitral valve prolapse; ASD, atrial septal defect; LVH, left ventricular hypertrophy; HTN, hypertension; RAD, right atrium dilation; RVH, right ventricular hypertrophy; TR, tricuspid regurgitation; PDA, patent ductus arteriosus; IUGR, intrauterine growth restriction.

^a^ Verhoeven et al., 2001; ^b^ Verhoeven et al., 2005; ^c^ Valayannopoulos et al., 2006; ^d^ Wamelink et al., 2008; ^e^ Fung et al., 2007; ^f^ Tylki-Szymańska et al., 2009; ^g^ Balasubramaniam et al., 2011; ^h^ Eyaid et al., 2013; ^i^ Jassim et al., 2014; ^j^Tylki-Szymanska A et al., 2014; ^k^ Leduc et al., 2014; ^l^ Al-Shamsi et al., 2015; ^m^ Banne et al., 2015; ^n^Lance H et al., 2016; ^o^ Lee-Barber et al., 2019; ^p^ Lafci et al., 2021; ^q^ Halabi et al., 2019.

The prenatal diagnosis of TALDO deficiency remains a challenge and is usually confirmed by gene analysis. Thus far, there is still no effective treatment for TALDO deficiency. Yet, early and accurate prenatal diagnosis can lead to a better outcome and can provide better aid for prenatal management, including fetal surveillance strategy and appropriate postpartum treatment, as was the case in the present study. In particular, a higher frequency of fetal surveillance with targeted ultrasound can help identify early signs of clinical manifestations (e.g., elevated MCA-PSV, cardiomegaly and placental thickness), which are important prognostic indicators. Most important of all, it is inseparable from the joint efforts of multi-disciplinary team. Currently, there is only one gene known to cause TALDO deficiency. Further studies are warranted to comprehensively characterize the genetic contributions.

In conclusion, our data suggests that TALDO deficiency is a pleiotropic disorder that should be considered when investigating a prenatal case with unexplained hepatosplenomegaly or fetal anemia. Although no specific treatment is currently available, targeted molecular analysis of the *TALDO1* gene in amniotic fluid or chorionic villi can be valuable in helping those suffering families to make informed reproductive choices.

## Data Availability

TThe original contributions presented in the study are included in the article/supplementary material, further inquiries can be directed to the corresponding author.
